# Strangulated Sliding Inguinoscrotal Hernia with a Gangrenous Bladder and Ileum

**DOI:** 10.7759/cureus.43028

**Published:** 2023-08-06

**Authors:** Vidhya Sree Sankaranarayanan, Madhusudhan Napa, Bhanumati Giridharan, Sandhya Palit, Nikhithaa Prabhuram

**Affiliations:** 1 General Surgery, Employees' State Insurance Corporation (ESIC) Medical College and Hospital, Chennai, IND

**Keywords:** herniorrhaphy, partial cystectomy, necrosed ileum, gangrenous bladder, strangulated slider hernia

## Abstract

An inguinal bladder hernia (IBH) is a common ailment in males above 50 years of age, with serious consequences of strangulation, if neglected. It is highly uncommon to have a strangulated inguinal hernia and bladder gangrene. This case reports a strangulated sliding inguinoscrotal hernia with a gangrenous bladder and ileum. We present a case of a 75-year-old man, presenting to the emergency room, with complaints of abdominal pain, distension, and absolute constipation. Examination revealed a large, firm, tender left-sided irreducible inguinal hernia. X-ray showed small bowel obstruction. Intraoperatively, a hernia sac was found with a gangrenous ileum as a slider along with a gangrenous fundus of the urinary bladder. Gangrenous segments were removed, and herniorrhaphy and bladder wall defect repair were performed. Even though a bladder can be involved in inguinal hernias, it is very rarely diagnosed preoperatively. In our case, there were no urinary symptoms, and the symptoms of strangulation outweighed bladder involvement. In any elderly patient with a giant hernia, a bladder entrapment should be ruled out with a strong index of suspicion. Failure to do so may result in complications after surgery. Hence, we conclude that it is better if all patients with long-standing giant hernias have a computed tomography (CT) prior to surgery.

## Introduction

An inguinal hernia is a frequent ailment that affects about 6% of the elderly population. While bladder inguinal herniation is not uncommon, the bladder is involved in only 1% to 3% of all inguinal hernias [1]. A scrotal cystocele was first defined by Levine in 1951 as a huge or enormous inguinal hernia containing a significant portion of the bladder. In elderly obese men over 50, the incidence may rise to 10%, particularly if there is an extended history of hernias [1,2]. The most prevalent and serious consequence of hernias is strangulation due to high morbidity and mortality rate. The prevalence of a strangulated inguinal hernia (SIH) is 1.3% overall and is more prevalent at the extremes of life [1-3]. A sliding hernia occurs when an internal organ composes a portion of the wall of the hernia sac. The bladder and colon are the two viscera that are most frequently affected. Failure to identify the visceral component of the hernia sac prior to bowel or bladder injury is the major cause of concern for a sliding hernia [1-4]. Due to the extensive collateral blood supply of the urinary bladder, gangrene of the bladder itself is a fairly uncommon clinical phenomenon [1-5]. It is highly uncommon to have a SIH and bladder gangrene. The main goals of this report are to briefly review previously reported cases and add one more case of a strangulated sliding inguinoscrotal hernia with a gangrenous bladder and ileum to the literature, highlighting some unique findings of this case.

## Case presentation

Our patient, a 75-year-old male, had a left inguinoscrotal swelling for 12 years. He had no complaints as the hernia was reducible for 12 years. The patient had consistently declined to have surgery. He arrived at our emergency department complaining of terrible cramping, stomach discomfort, and distension, which had been going on for the previous five days. During this time, he did not have a bowel movement or passed any flatus. He restricted his intake to mere sips of water for the past five days. He had absolutely no urinary complaints. Physical examination revealed a moderately nourished man in severe distress from abdominal pain with a centrally distended abdomen. He was severely dehydrated and pale and gave the impression of being seriously ill. The patient’s blood pressure was 90/60 mmHg, pulse rate was 128/min, and temperature was 98.8 °F. Abdominal examination revealed a large, hard, and tender mass in the left inguinal region extending into the scrotum. The swelling measured 7 x 5 inches and was fixed and irreducible. There was guarding and rigidity as per abdominal examination. The left cord structures were palpable at the base of the penis, and the left testis was palpable vaguely at the tip of the swelling; right-sided cord structures and testis were normally palpable. No bowel sounds could be heard. The prostate gland was enlarged, and the rectum was vacant and free of any tumors.

The results of the tests were as follows: white blood cell count was 30,000 per cubic millimeter, hematocrit was 47%, and hemoglobin was 14.2 gm/dl. According to the differential count, there were 72% segmented neutrophils, 8% non-segmented neutrophils, 10% lymphocytes, 9% monocytes, and 1% eosinophils. Amber and hazy urine with a specific gravity of 1.023 and a small amount of albumin was present. In the high-power field, there were 50 to 60 red blood cells and 12 to 14 white blood cells. Blood urea nitrogen concentration was 17%. A considerable amount of gaseous distention of the small bowel loops with a step-ladder pattern was seen on an abdominal roentgenogram.

**Figure 1:**
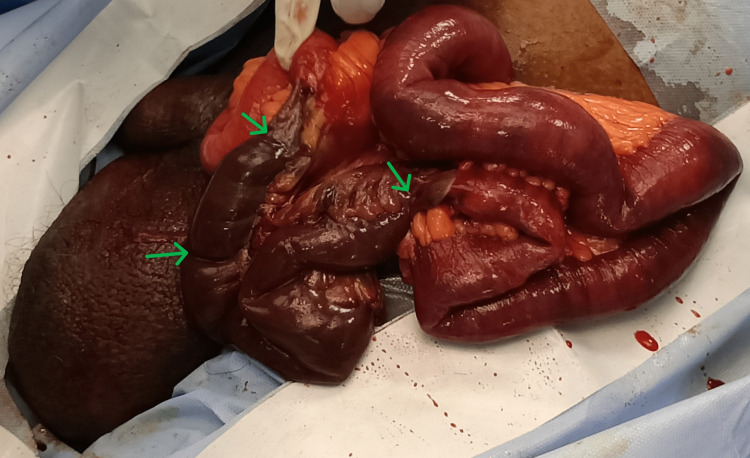
Bowel loop measuring 100 cm. The green arrows point to the multiple constriction rings on the grangrenous ileum.

The patient was admitted, given intravenous crystalloid fluids for resuscitation, and was prepared for an emergency surgery. A left inguinoscrotal exploration (later extended to a midline laparotomy), under general anesthesia after receiving informed written consent, was done, and a mass of stench-filled tissues was delivered from the inguinoscrotal region. A hernial sac was found and isolated on the posteromedial side of the mass. When the sac was opened, foul-smelling, dark crimson fluid was discovered inside. There was an indirect defect (via superficial inguinal ring) through which the gangrenous ileum had herniated as a slider. The loop, which was about 100 cm long and black and gangrenous, was the incarcerated ileal section with numerous constriction rings (Figure 1). After performing a resection of the gangrenous segment of the ileum, an end-to-end ileoileal anastomosis was done to restore bowel continuity. The rest of the bowel was found to be normal.

**Figure 2:**
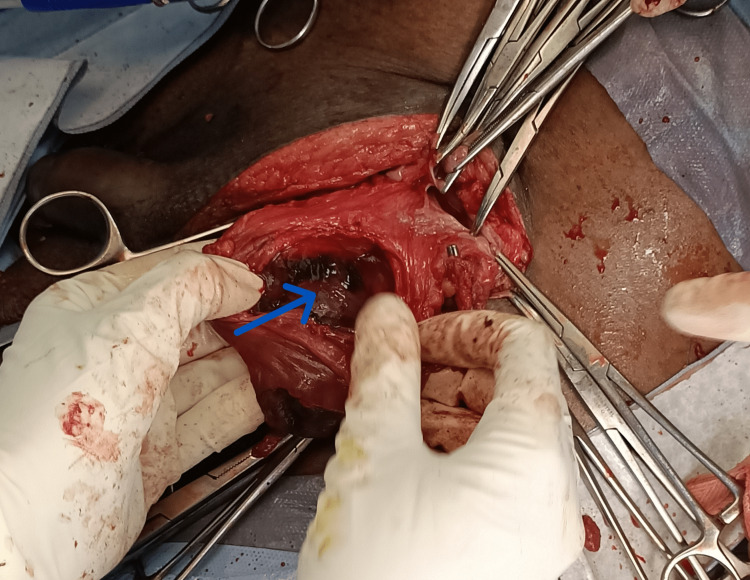
Fundus of the bladder was gangrenous across all of its layers, with gangrene measuring around 5 x 2 cm in circumference. The blue arrow indicates the defect after the removal of gangrenous fundus of the urinary bladder.

A 5 cm x 2 cm mass of tissue that was adherent to and indistinguishable from cord components was felt medial to the sac. The tissue had a bad odor and was dark red in color with patches of black gangrene. The presence of a Foley catheter bulb confirmed that the mass, which had a fluctuant appearance, was the fundus of the urinary bladder along with the prevesical fat. When the bladder's fundus was opened, it was discovered to be gangrenous across all of its layers, with gangrene measuring 5 x 2 cm in circumference. The bladder margins were clipped, the gangrenous section was removed, and the bladder wall defect was repaired using two layers of chromic catgut suture, principally over a suprapubic 20F Foley catheter (Figure 2). Due to strong adhesions between the testis and the gangrenous ileum, an ipsilateral orchiectomy was performed. The bladder’s interior was examined before being sealed and was confirmed to be in good condition.

Herniorrhaphy (posterior wall repair, with prolene used to suture the conjoint tendon with the inguinal ligament) was used to treat the hernial defect, and the skin was closed without drainage. A mesh repair was not attempted because it was a gangrenous bowel. The urine that drained from the suprapubic catheter (SPC) was grossly clear but contained microscopic red blood cells. Following surgery, the hourly urine production was observed in order to prevent clogging of the Foley catheter. The catheter was not irrigated but was frequently examined by the medical team. In the first few days following the surgery, the patient did well. The patient received cefotaxime and metronidazole for seven days while remaining afebrile.

He was discharged afterward, with an SPC with a weekly follow-up on outpatient department (OPD) basis. Up to the 31st surgical day, the urine output was adequate. The patient first felt urine dripping from the inguinal wound on the fortieth postoperative day. After a thorough examination, it became clear that the patient had a vesico-cutaneous fistula that was draining urine through the side of the inguinal scar. It was confirmed with a contrast-enhanced computed tomography (CT) scan of the abdomen and pelvis. A second surgery was performed on the patient with the help of a urologist by reopening the lower abdomen midline the laparotomy site. The bladder was resected in a partial cystectomy containing the internal opening of the fistula, and reinforcement of the posterior wall was done. The part of the inguinal skin containing the external opening of the fistula was excised, and routine skin closure was done.

The patient was on antibiotic treatment with amoxicillin for seven days while postoperatively being afebrile. His urine production was routinely measured, and the results were satisfactory. His prostate-specific antigen (PSA) test results were within the expected range. Up to the 21st day, the SPC was used to administer bladder wash twice daily. The SPC was clamped, and the patient was observed until the urethral catheter drained urine, after which the SPC was removed. Up to the patient's discharge, there were no new issues with the catheter sites. On the 34th postoperative day, the urethral catheter was clamped, and the patient was instructed to hold off on urinating until he felt his bladder was full. The patient had bladder training for seven days before having the urethral catheter withdrawn and being able to void properly. He was released on the 49th day following the second surgery. At his two-week follow-up appointment, his only complaint was that he had noted an increased frequency of urination, for which he was given instructions on bladder training exercises.

## Discussion

A hernia is defined as an abnormal protrusion of an organ or tissue through a fascial defect in its surrounding walls [2-18]. Although a hernia can develop anywhere on the body, it most frequently affects the abdominal wall, in 75% of the patients, the inguinal area [2-5]. A fatal complication of a hernia is strangulation causing compromised blood flow to its contents. The narrow neck of the hernia prevents either venous drainage, arterial blood flow, or both to the hernia sac's contents [3-7]. Aging also increases the risk of strangulation and the requirement for hospitalization [4-15]. The inferior pelvis houses the bladder, the end reservoir for urine. Due to the extensive collateral circulatory network of the bladder, artery ligation or damage has no detrimental impact on the bladder [5-9]. It is not uncommon for the bladder to be involved in inguinal hernias. Weakness in the bladder and abdominal walls is a contributing factor in inguinoscrotal bladder hernias' pathogenesis. Increased intra-abdominal pressure during micturition due to the presence of bladder outlet obstruction, such as prostatic enlargement (as in our instance) or urethral stricture, favors herniation [8-16]. Herniation can also be caused by a weak pelvic floor or by space-occupying pelvic masses. In women, pelvic inflammatory weakness, which results in adhesions between the peritoneum and bladder, has been identified as a predisposing factor. In addition, herniation may occur as a result of trauma or surgical trauma and obesity [8-12].

The majority of inguinal bladder hernias (IBHs) (77%) are discovered intraoperatively, 7% preoperatively, and 14% postoperatively during complications [5-13]. The majority of sliding hernias involving the bladder are discovered intraoperatively, either during the hernia sac dissection or following an unintentional cystotomy [6-18]. The most frequently reported type of IBH, which is also present in our case, is a para-peritoneal hernia [6-15]. In this form, the bladder is positioned medially or posteriorly to the hernial sac. The serous covering on the exterior of the bladder hernia is the same peritoneum that lines the inner portion of the sac [7-13]. As a sliding hernia, the bladder follows the sac and lies external to the sac, with the peritoneum just covering the exterior wall. In intraperitoneal hernias, the peritoneum of the sac entirely encloses the bladder, which is located inside of it. These hernias often have a big size and are rarely strangulated [7-17].

When a bladder hernia is suspected based on the patient's medical history, a voiding cystogram should be done [9-12]. Cystography typically reveals a bladder that resembles a dumbbell. Magnetic resonance imaging, CT, and ultrasonography are some of the investigations for scrotal cystoceles. Imaging helps with diagnosis and surgical planning by allowing the identification of anatomical anomalies and potential issues [10-17].

Following a normal inguinal hernia repair, with or without mesh, if a bladder hernia is unintentionally discovered during herniorrhaphy, recommendations include either reducing the bladder back to its anatomic place or selectively resecting the herniated bladder component [10-18]. When an injured bladder is discovered after surgery for an incarcerated or strangulated groin hernia, conversion to midline laparotomy is advised, especially in the emergency scenario as documented in our study. If the bladder wall is necrotic or irreparable, partial cystectomy is advised [7-11]. Inadvertent surgical damage during any herniorrhaphy may be reduced with bladder decompression via urethral catheterization [11-17].

Due to adhesions, the repair of a major bladder herniation may need intestinal resection and orchidectomy. In addition, abdominal compartment syndrome, wound dehiscence, and hernia recurrence might make the situation more challenging [12-15]. Cystolithiasis, vesicoureteric reflux, sepsis, unilateral or bilateral ureteric blockage, renal failure, strangling with subsequent ischemia of the bladder wall as seen in our case, and bladder rupture are complications of an inguinoscrotal bladder herniation [12-14].

## Conclusions

Diagnosing an IBH is a challenge faced by every surgeon, especially when confronted with a patient who is hemodynamically unstable. Relying exclusively on clinical signs and laboratory investigations to take up a patient for surgery will not suffice. Any patient with a giant long-standing inguinoscrotal hernia, with or without urinary symptoms (or renal impairment including acute kidney injury as was present in our patient), should be considered to have urinary bladder as a content, unless proven otherwise. The experience gained from the management of this patient has led us to recommend the following: First, it should be made a mandatory practice to do a contrast CT before such a patient is taken up for elective or emergency surgery. Second, in patients with renal impairment, a low-dose contrast should be considered with peri-procedural fluid resuscitation. If the bladder was found to be involved and/or repaired, temporary urinary diversion by means of suprapubic catheterization should always be done until the patient is bladder-trained satisfactorily.
